# Efficacy of therapeutic intervention with NanoBEO to manage agitation and pain in patients suffering from severe dementia: a pilot clinical trial

**DOI:** 10.3389/fphar.2024.1417851

**Published:** 2024-08-01

**Authors:** Damiana Scuteri, Martina Pagliaro, Isabel Mantia, Marianna Contrada, Loris Pignolo, Paolo Tonin, Pierluigi Nicotera, Giacinto Bagetta, Maria Tiziana Corasaniti, the Pilot BRAINAID Trial investigators

**Affiliations:** ^1^ Department of Health Sciences, University “Magna Graecia” of Catanzaro, Catanzaro, Italy; ^2^ Preclinical and Translational Pharmacology, Department of Pharmacy, Health Science and Nutrition, University of Calabria, Cosenza, Italy; ^3^ Regional Center for Serious Brain Injuries, S. Anna Institute, Crotone, Italy; ^4^ The German center for Neurodegenerative Diseases (DZNE), Bonn, Germany

**Keywords:** NanoBEO, agitation, dementia, behavioural and psychological symptoms of dementia, pain, pilot clinical trial, BRAINAID, NCT04321889

## Abstract

**Background:**

An estimated 57.4 million people live with dementia worldwide, with the social burden of the disease steadily growing. Despite the approval of lecanemab and ongoing trials, there is still a lack of effective and safe treatments for behavioral and psychological symptoms of dementia (BPSD), which affect 99% of patients. Agitation is one of the most disabling BPSD, with a cross-sectional prevalence of ≥50% in nursing homes, and refers to help-seeking behavior in response to various sources of discomfort, among which pain is a crucial component.

**Methods:**

This pilot phase of the BRAINAID (NCT04321889) trial aimed to assess the effectiveness of the patented nanotechnological device NanoBEO in older (≥65 years) people with severe dementia. This randomized placebo-controlled trial, with quadruple masking that involved all operators and participants, followed the SPIRIT and CONSORT statements. A total of 29 patients completed the trial. The patients were randomly allocated in a 1:1 ratio to the NanoBEO or placebo group, and the corresponding product was applied on both arms once daily for 4 weeks, with a 4-week follow-up period. The primary endpoint was efficacy against agitation. The secondary endpoints were efficacy against agitation at follow-up and efficacy against pain. Any adverse events were reported, and biochemical analyses were performed.

**Results:**

The NanoBEO intervention reduced the frequency (28%) and level of disruptiveness of agitated behaviors. The effect on frequency was statistically significant after 2 weeks of treatment. The efficacy of NanoBEO on agitated behaviors lasted for the entire 4-week treatment period. No additional psychotropic drugs were prescribed throughout the study duration. The results after 1 week of treatment demonstrated that NanoBEO had statistically significant analgesic efficacy (45.46% improvement in pain intensity). The treatment was well tolerated.

**Discussion:**

This trial investigated the efficacy of NanoBEO therapy in managing agitation and pain in dementia. No need for rescue medications was recorded, strengthening the efficacy of NanoBEO in prolonged therapy for advanced-stage dementia and the usefulness of the intervention in the deprescription of potentially harmful drugs. This study provided a robust rationale for the application of NanoBEO in a subsequent large-scale pivotal trial to allow clinical translation of the product.

**Clinical Trial Registration:** ClinicalTrials.gov, identifier NCT04321889.

## 1 Introduction

The medical and social burden of dementia is steadily growing. The disease affects approximately 57.4 million people worldwide, with the number estimated to triple by 2050, and more women are living with dementia than men (women:men ratio of 1.67) (GBD, 2022). The recent approval by the Food and Drug Administration (FDA) of lecanemab for early Alzheimer’s disease (AD) (Doggrell, 2024) renewed interest in the β-amyloid (Aβ) theory. The lack of disease-modifying drugs may lead to inappropriate treatment of the behavioral and psychological symptoms of dementia (BPSD), without much supporting evidence for the efficacy or safety of interventions (Scuteri et al., 2021b). Ongoing clinical trials are investigating pathophysiology-based disease-modifying medications, from small molecules such as simufilam (Wang et al., 2023) (NCT05575076; NCT04994483) to passive immunotherapies such as the novel remternetug directed against a pyroglutamated form of Aβ (NCT05463731). Despite progress, the main target remains the stage of amnestic mild cognitive impairment (aMCI) and prodromal AD (Huang et al., 2023). Potential therapeutic mechanisms involve neuroprotection, anti-inflammation, cognitive enhancement, neuropsychiatric control and, in the frame of the latter actions, drugs that can induce autophagy are promising candidates to reduce neurodegeneration (Chu, 2006; Metaxakis et al., 2018). However, clinically useful autophagy inducers with measurable effect on autophagy that are safe and can cross the blood–brain barrier deserve further investigation (Corasaniti et al., 2024).

With almost all patients (99%) experiencing at least one symptom (Pinyopornpanish et al., 2022), management of BPSD is challenging, mainly in the advanced stages of the disease. BPSD are presented differently across individuals and with various degrees of severity, and are associated with poor outcomes (Cerejeira et al., 2012). Moreover, it is important to point out the need for consideration and inclusion in clinical trials of several, less frequently occurring, phenotypes such as posterior cortical atrophy (Bejanin and Villain, 2024). BPSD usually occur chronically in a fluctuating pattern, and patients present at least one symptom at subsequent assessments; thus, BPSD are associated with longitudinal cognitive decline (Burhanullah et al., 2020). Recently, the presence of BPSD was linked to gray and white matter lesions, with multiple correlations observed in patients with hyperactivity using single-photon emission computed tomography (SPECT) and T1-weighted magnetic resonance imaging (MRI) (Nakase et al., 2023). BPSD are often under-recognized and early depression and mild behavioral impairment (MBI) can be prodromal to cognitive impairment (Ismail et al., 2017); however, this knowledge did not spur effective and safe pharmacological therapies (Scuteri et al., 2021b). The prospective, population-based, longitudinal Cardiovascular Health Study-Cognition Study demonstrated a high risk of developing MCI in up to 19.7% of the people who experience moderate-to-high depressive symptoms (Barnes et al., 2006). In particular, the results by the European Alzheimer’s Disease Consortium in 2,808 patients with dementia demonstrate a consistent occurrence of hyperactivity, affective symptoms, psychosis, and apathy, with the latter two symptoms correlated with the use of cholinesterase inhibitors and dementia severity, respectively (Aalten et al., 2008). The prevalence of some BPSD was estimated in people with dementia (PwD; n = 587) and non-affected counterparts (n = 2,050) in a population-based longitudinal study of ageing; the study highlighted that all BPSD (except sleep disorders) occurred more often in PwD and that certain symptoms—psychosis/apathy, depression/anxiety, irritability/persecution, and wandering/sleep problems—co-occurred (Savva et al., 2018). All these findings point to the burden of BPSD.

Agitation is one the most common and disabling BPSD that mainly affects PwD at moderate to severe stages. A cross-sectional prevalence of over 50% in nursing homes has been reported, with at least one item of the Cohen-Mansfield Agitation Inventory (CMAI) presented weekly in 75.4% of the cases (Testad et al., 2007). Moreover, a high correlation has been found between the CMAI score and stage of dementia (Spearman rho = 0.421, *p* = 0.000); patients who received psychoactive medication had a higher mean CMAI score (39.9, SD 13.1), and the use of psychotropic drugs correlated with the stage of dementia (Testad et al., 2007). Dementia is often underdiagnosed and co-occurring with agitation and inappropriate treatment (Scuteri et al., 2021e), as evidenced in data collected from a sample of 1,163 patients, of whom 81% presented with dementia, 72% experienced clinically relevant BPSD, and 75% received psychotropic medications (Selbæk et al., 2007).

Agitation can be defined as help-seeking behavior in response to various sources of discomfort, among which pain is a crucial component (Husebo et al., 2014). PwD are usually affected by age-related comorbidities that cause chronic pain, which remains underdiagnosed because of the lack of self-report measures (Sampson et al., 2015); in fact, approximately 80% of the patients with dementia in nursing homes experience pain (Achterberg et al., 2013). Agitation treatment relies on atypical antipsychotics, among which risperidone has received approval and, on 11 May 2023, the FDA announced the supplemental approval of brexpiprazole for the treatment of agitation associated with dementia (Cummings et al., 2024); notably, these drugs potentially increase the risk of death due to cardio-cerebrovascular accidents (Schneider et al., 2005; Cummings et al., 2024). In people with moderate-to-severe, but not mild, AD, memantine exerts a small clinical effect (McShane et al., 2019), while other, off-label, drugs, such as antidepressants and benzodiazepines, worsen cognitive decline and enhance the risk of harmful falls (Harris and Lykina, 2022).

In view of the possible role of non-pharmacological therapies in BPSD (Davison et al., 2024), the correlation between pain and agitation, and the confirmed priority of analgesia in BPSD management, appropriate and integrated measures to target pain (Corbett et al., 2014) are needed to safely treat agitation. Although aromatherapy with *Melissa officinalis* essential oil improves the CMAI score (Ballard et al., 2002), its superiority to placebo or donepezil has not been demonstrated (Burns et al., 2011). Likewise, the efficacy of aromatherapy with *Lavandula angustifolia* essential oil is controversial (Holmes et al., 2002; Lin et al., 2007; O'Connor et al., 2013; Moorman Li et al., 2017; Zalomonson et al., 2019). Managing the robust scent of essential oils poses considerable challenges when designing real-world double-blinded clinical trials; this and other methodological biases have hindered any definite conclusions regarding the efficacy of aromatherapy in dementia (Forrester et al., 2014), similar to the situation regarding the application of nutraceuticals in other neurodegenerative diseases such as glaucoma (Scuteri et al., 2020). Strong preclinical evidence of efficacy has been reported for the essential oil of bergamot (BEO; *Citrus bergamia* Risso et Poiteau) in models of pain relevant to clinical conditions (Scuteri et al., 2021c). Efficacy has been shown after continuous administration (Hamamura et al., 2020) and via inhalation (Scuteri et al., 2018; Scuteri et al., 2022d) and transdermal application (Scuteri et al., 2022c), useful for aromatherapy. Furthermore, BEO exerts anxiolytic activity devoid of the sedative effects typical of benzodiazepines (Rombolà et al., 2020). The nonvolatile fraction of BEO, representing the 4%–7% of total, contains furocoumarins, e.g., bergapten (Mondello et al., 1993; Dugo et al., 2000), that can induce phototoxic reactions of the skin caused by the photoactivation of bergapten due to ultraviolet light (Zaynoun et al., 1977). BEO was delivered in a bergapten-free form to avoid phototoxicity, the only documented side effect according to the assessment report of the European Medicine Agency (EMA) (13 September 2011 EMA/HMPC/56155/2011 Committee on Herbal Medicinal Products [HMPC]). In fact, furocoumarin-free BEO was engineered in a nano-size delivery system, based on solid lipid nanoparticles, and formulated as an odorless cream known as NanoBEO (Scuteri et al., 2021a).

In the frame of the pilot phase of the BRAINAID trial (NCT04321889) (Scuteri et al., 2021d), the aim of the present clinical study was to assess the effectiveness of NanoBEO on agitation as primary endpoint and pain as secondary endpoint in PwD at the severe stage. To the best of our knowledge, this is the first trial allowing double-blindness, and also quadruple masking, due to the entrapment of BEO aroma, to investigate the effects of the formulation on agitation in severe dementia.

## 2 Materials and methods

The aim of the present pilot clinical study was to assess the effect of NanoBEO on agitation in older people with severe dementia. NanoBEO was prepared as an odorless cream, indistinguishable from the placebo cream, using nanotechnology to load BEO. This pilot trial was designed as a randomized, quadruple-blind placebo-controlled trial (NCT04321889) and was registered in ClinicalTrials.gov on 23 March 2020, to assess the efficacy of furocoumarin-free BEO loaded in a nanocarrier delivery system in the treatment of agitation in older individuals with severe dementia. This study was approved by the Calabria Region Ethics Committee (protocol No. 352, first version; 21 November 2019). The protocol and trial followed the Standard Protocol Items: Recommendations for Interventional Trials (SPIRIT) (An-Wen et al., 2013) and the Consolidated Standards of Reporting Trials (CONSORT) (Kenneth et al., 2010) guidelines.

This trial was designed as a prospective, single-center, exploratory interventional study without a drug. The coordination center was Sant’Anna Institute, the regional center of care and research for severe brain injuries, which specializes in motor and cognitive treatment and rehabilitation of patients with neurological diseases. The data were collected from nursing homes in Southern Italy, which were recruited for this study. This clinical trial was intended as a pilot study before a subsequent large-scale, adequately powered, pivotal study.

The primary endpoint was the clinical effectiveness of NanoBEO in a 4-week treatment of agitation in patients with severe dementia. The secondary endpoints were changes in agitation in a follow-up period after the end of the intervention and the clinical efficacy of NanoBEO against pain in patients with severe dementia.

### 2.1 Eligibility criteria

Consecutive patients with a diagnosis of dementia were enrolled according to the following inclusion criteria: 1) mini-mental state examination (MMSE) score ≤12 and 2) provision of informed consent by a legal representative.

Patients were reported to receive quetiapine and promazine for psychotic, aggressive disorders and mirtazapine, paroxetine, trazodone and alprazolam, bromazepam, delorazepam or clonazepam for depressive and anxious manifestations (also concurrently in some cases) and, occasionally, acetaminophen. One case of use of haloperidol, one of zolpidem and one of gabapentin were recorded at baseline. The patients were allowed to receive authorized concurrent therapies for the treatment of agitation (risperidone for aggressive behavior). Therapies for the treatment of other chronic comorbidities—such as drugs for the treatment of hypertension or diabetes, for gastric protection, anti-inflammatories, and antibiotics—were allowed. A legal representative of the patient was informed about the study and provided a consent form, which was collected by healthcare operators.

Patients with a clinical history of disabling neurological or psychiatric diseases (including Parkinson’s disease, stroke, cerebral hemorrhage, delirium, and psychosis) were excluded.

### 2.2 Treatments and chemicals

For the production of NanoBEO according to the patent specifications (request and concession number 102019000013353) (Scuteri et al., 2021a), BEO was kindly supplied by Capua 1880 S.r.l. (Campo Calabro, Reggio Calabria, Italy). The chemical composition of BEO and the percentage ranges of its most abundant components are presented in Table 1.

**TABLE 1:** Percentage ranges of the main components of the essential oil of bergamot (BEO).

Chemical substance	Ranges (%)
α-Pinene	0.7–2.0
Sabinene	0.5–2.0
β-Pinene	5.0–10.0
Limonene	30.0–50.0
γ-Terpinene	6.0–18.5
Linalool	6.0–15.0
Linalyl acetate	23.0–35.0
Geranial	< 0.5
Geranyl acetate	0.1–0.7
Cariophyllene	0.2–0.5

### 2.3 Collection of biological specimens

Biological specimens for biochemical analyses (azotemia and serum creatinine, creatine phosphokinase [CPK], and transaminase levels) were collected before treatment and weekly during treatment and follow-up, in accordance with standard care practices.

### 2.4 Treatment schedule and procedure

**FIGURE 1:**
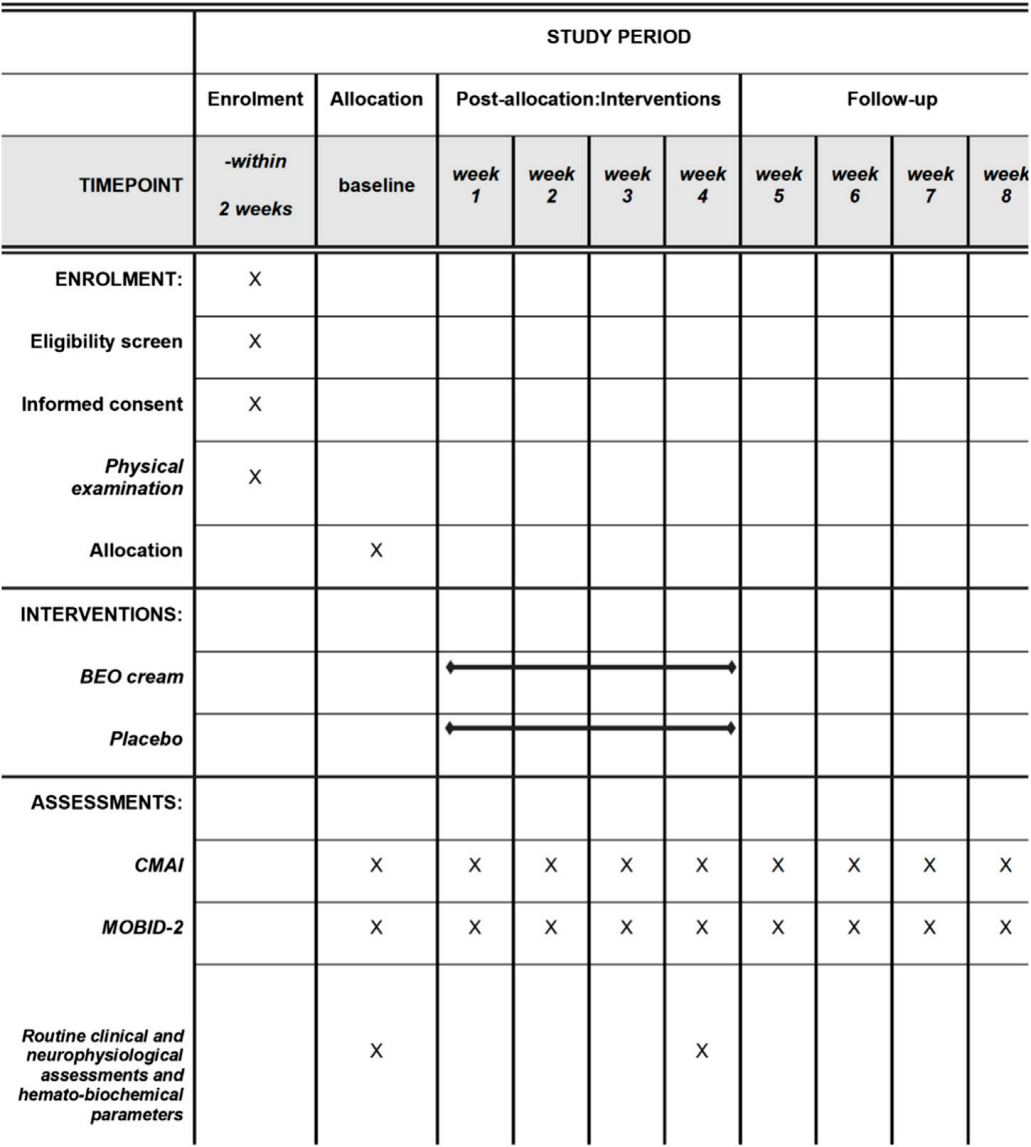
Schedule for patient enrollment, treatment administration, follow-up, and related outcome measures. CMAI, Cohen-Mansfield Agitation Inventory; MOBID-2, Mobilization–Observation–Behavior–Intensity–Dementia test.

The patients who met all the inclusion criteria and none of the exclusion criteria were enrolled in the study and randomized in a 1:1 allocation ratio to the active intervention (NanoBEO) or the placebo group. To avoid any bias associated with sequence generation, the design involved no blocking (e.g., incomplete randomization). The allocation randomization codes were obtained using the random number generator in Microsoft Office Excel 2010 (Milan, Italy). No member of the trial who administered the treatments or analyzed the data had access to the codes until the end of the trial, to ensure adequate allocation concealment and prevent performance and detection biases. The operators who independently recruited patients were different from those who generated the allocation sequence and those who assigned participants to the two arms. Healthcare personnel, patients, outcome assessors, and data analysts were blinded to the assignment to interventions, allowing quadruple masking. To guarantee security and data quality, two operators performed double data entry and the collection and maintenance of patient information was only handled by the administration staff of the clinical trial unit at the coordinating center; this strategy was selected to protect confidentiality before, during, and after the trial. After randomization (T0), all patients in both groups were administered one application containing a dose of 1 g of active cream or placebo cream on each arm once daily for 4 weeks between 8:00 and 10:00 a.m. A single dispenser covered the entire 4-week treatment. The procedure was completed in approximately 2 min. The packaging was identical for NanoBEO and the placebo and the two products were indistinguishable in terms of appearance, texture, and scent. After signing the informed consent form to participate in the study, the patients at T0 were assessed for baseline clinimetric variables using MMSE, CMAI, and the Italian Mobilization–Observation–Behavior–Intensity–Dementia (I-MOBID2) pain scale. We also collected anamnestic data, which included significant medical events in the last 30 days, administered drugs or changes in therapies, and any complementary therapies. Administration of CMAI and MOBID-2 was repeated weekly for 4 weeks (T1A first week, T1B, second week, T1C third week, T1D last week of treatment) and again weekly for another 4 weeks (T2A first week, T2B, second week, T2C third week, T2D last week of follow-up). A schematic representation of the study procedure is shown in Figure 1. Adverse events were recorded on a specific form to assess the following aspects: symptom severity (mild, moderate, or severe); correlation with treatment administration (suspected/not suspected); duration (start and end or if present at the time of the final evaluation); and serious adverse events.

This trial recruited patients with difficulties in communication, who resided in nursing homes; NanoBEO and the placebo cream were topically administered by healthcare operators to ensure that the specified interventions were adhered to. The healthcare operators were asked to retain each empty dispenser. To avoid attrition bias due to deviations from the protocol by excluding patients from the analysis who failed to follow to the protocol, we used an intention-to-treat approach, declaring drop-outs and including patients up to trial discontinuation.

### 2.5 Outcome measures

**FIGURE 2:**
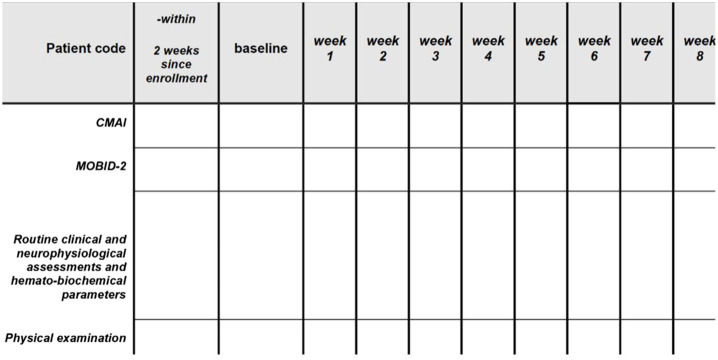
Data collection form.

The outcome measures were the CMAI scores for agitation and I-MOBID2 scores for pain. All the raters and responders (as the CMAI is assessed by a researcher/operator who interviews a caregiver) underwent training, in which descriptions of the tools used were provided to guarantee correct execution of the assessment and inter-rater reliability. As per the a priori-set protocol of the study, the same rater performed the baseline assessment and the evaluations during treatment administration and follow-up. As reported above, pain assessment in PwD with communicative difficulties is complex and observational pain scales have been devised for patients with severe dementia and compromised communicative abilities. The CMAI assessment was completed in 20 min, while the I-MOBID2 assessment required approximately 5–6 min. A template of the data collection form, which was then copied into Excel format, is shown in Figure 2.

### 2.6 Statistical analysis

The patients were considered in pain when the I-MOBID2 items or overall pain intensity were scored ≥3 and with a CMAI score for agitation ≥39. No sample power calculation was performed because this study was not interventional with new drugs; however, the pilot phase of the BRAINAID clinical trial for which a statistical analysis plan (SAP) with sample power calculation has been provided (Scuteri et al., 2021d). The statistical differences between the two groups for baseline patient characteristics were assessed using the Student’s t-test and Mann–Whitney *U* test (MWU). The results are presented as the median, with 95% confidence intervals (CI) and interquartile range (IQR). The statistical differences in individual medians were assessed using two-way ANOVA for repeated measures, followed by Tukey’s multiple comparison test. All analyses were performed using Microsoft Office Excel 10 and GraphPad Prism 6.0 (GraphPad Software by Dotmatics, CA). Values of *p* ≤ 0.05 were considered statistically significant.

## 3 Results

### 3.1 Characteristics of the patients

Thirty-one patients were screened for eligibility. One patient was transferred to another nursing home and was excluded. Thus, the study included 30 participants from eight nursing homes. Of these patients, 14 were allocated to the NanoBEO arm and 16 to the placebo arm. The baseline characteristics of the two groups were similar, except for a trend for higher, but not significantly different, baseline I-MOBID2 scores in the NanoBEO group. The mean age of all participants was 86.83 ± 6.87 (standard deviation [SD]) years, with a mean age of 87.50 ± 6.68 and 86.07 ± 7.26 for the patients allocated to the placebo and NanoBEO arms, respectively. Most patients were women, with one male patient per group, in agreement with descriptions of the sex distribution and prevalence of the disease and considering the small sample size. Apart from one patient who was subsequently allocated to the placebo group, all the patients were prescribed at least one psychotropic medication, with one patient receiving only dihydrocodeine with psychotropic action. The baseline characteristics of the patients are presented in Table 2.

**TABLE 2:** Age and baseline assessment of agitation and pain in the patients enrolled in the trial and subsequently allocated to the NanoBEO and placebo groups.

Baseline characteristics	NanoBEO group (n = 14)	Placebo group (n = 16)	Statistical analysis: *p*-Value and 95% CI
Age (mean ± SD)	86.07 ± 7.26	87.50 ± 6.68	0.58 *t*-test
CMAI-frequency (F; median)	50.50	50.50	0.64 (−21.00; −11.00) MWU test
CMAI-disruptiveness (D; median)	43.00	43.00	0.43 (−14.00; −6.00) MWU test
I-MOBID2 (median)	5.5	3.00	0.17 (−3.00; 1.00) MWU test

SD, standard deviation; CI, confidence intervals; I-MOBID2, Italian Mobilization–Observation–Behavior–Intensity–Dementia; MWU, Mann–Whitney *U* test.

**FIGURE 3:**
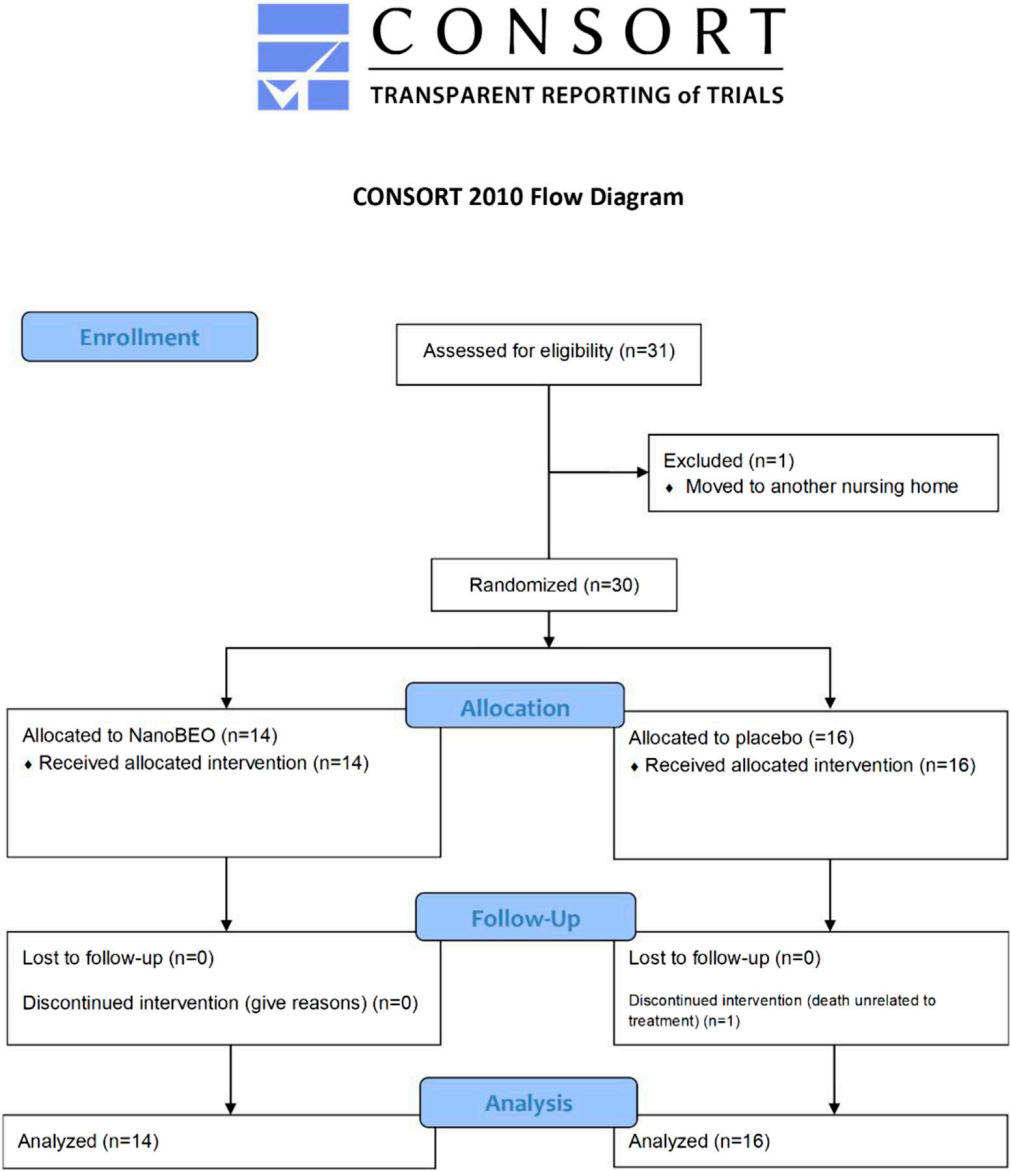
Consolidated Standards of Reporting Trials (CONSORT) flow diagram of progress through the various stages—enrollment, allocation, intervention, follow-up, drop-out, and analysis—of the pilot trial on the effectiveness and safety of NanoBEO in dementia.

Twenty-nine patients (96.67%) completed the intervention phase and follow-up. One participant (age, 92 years) in the placebo group died after having completed the first week of treatment; his death was attributed to cardiac and respiratory illness and was unrelated to the study treatment. Over the course of the study, no additional psychotropic drugs were prescribed as rescue medications for increased agitation. The analysis includes data from 16 patients in the placebo group for the first week and from 15 patients after the death of one patient. The processes related to enrollment, group allocation, follow-up, and analysis are reported in the CONSORT flow diagram in Figure 3.

### 3.2 Efficacy of treatment on agitation

**FIGURE 4:**
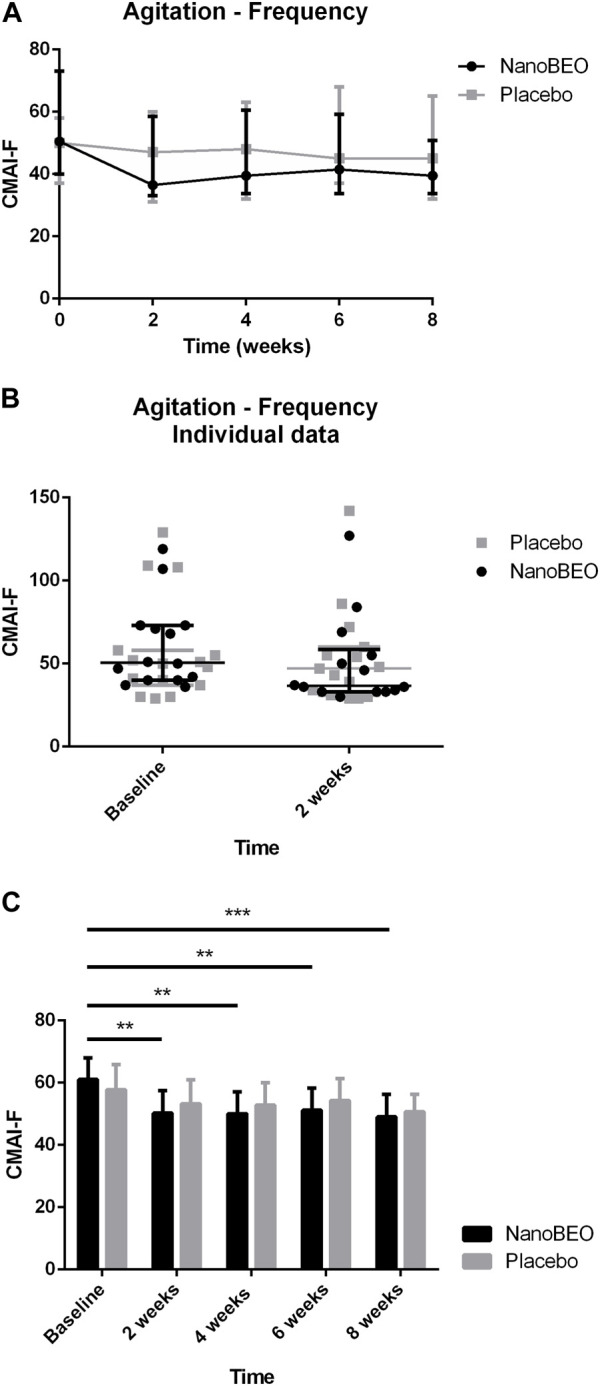
Efficacy of NanoBEO on the frequency of agitation based on the CMAI-F scores during treatment and follow-up **(A)** and as individual data at baseline and after 2 weeks of treatment **(B)**; data are expressed as the median + interquartile range (IQR). Patients allocated to the NanoBEO group experienced a reduction in CMAI-F scores than did patients allocated to the placebo group. **(C)** Statistically significant differences regarding NanoBEO efficacy were observed for all time points *versus* baseline (data are expressed as the mean ± SEM; time factor ****p < 0.0001; participant matching ****p < 0.0001; NanoBEO: 2, 4, and 6 weeks **p < 0.05, 8 weeks ***p < 0.001). *p values <0.05 are considered to indicate statistical significance. n: NanoBEO = 14, placebo = 16. SEM, standard error of the mean.

**FIGURE 5:**
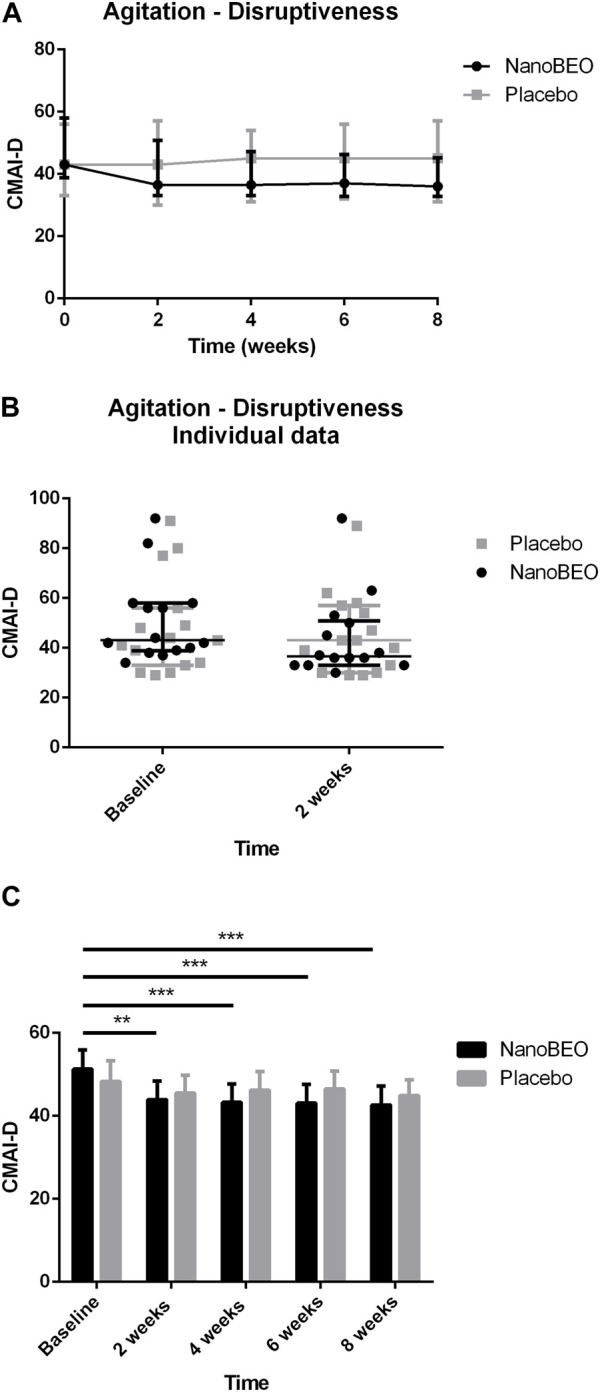
Efficacy of NanoBEO on disruptiveness of agitation based on the CMAI-D scores during treatment and follow-up **(A)** and as individual data at baseline and after 2 weeks of treatment **(B)**; data are expressed as the median + IQR. Patients allocated to the NanoBEO group experienced a reduction in CMAI-D scores than did patients allocated to the placebo group. **(C)** Statistically significant differences regarding NanoBEO efficacy were observed for all time points *versus* baseline (data are expressed as the mean ± SEM; time factor ****p < 0.0001; participant matching ****p < 0.0001; NanoBEO: 2 weeks **p < 0.01; 4, 6 and 8 weeks ***p < 0.001). *p values <0.05 indicate statistical significance. n: NanoBEO = 14, placebo = 16.

A reduction in both the frequency (Figures 4A–C) and level of disruptiveness (Figures 5A–C) of agitated behaviors, as assessed by the CMAI scores, were observed in the patients allocated to the NanoBEO group in comparison to those in the placebo group. The CMAI is a caregiver-rated questionnaire that consists of 29 items that examine behaviors associated with agitation; the scores range from 29 to 203, with significant agitation indicated at scores ≥39. The frequency of presentation of each behavior is rated on a seven-point scale based on assessments in the preceding 2 weeks. The frequency of occurrence of behaviors was rated as follows: never; less than once a week; once or twice a week; several times a week; once or twice per d; several times per d; and several times per h. Each behavior can be represented by a wide spectrum of impairments; thus, the raters and respondents were provided with a detailed description of behaviors. Correct execution of the test was explained, emphasizing that the closest related item and similar behavioral indicators should be included, even when these were not exactly reported in the behavioral descriptors. To assess the level of agitation, the assessor/interviewer conducted an interview with the caregiver/respondent familiar with the patients, who was provided with a copy of the scale several days before. On the day of assessment, the interviewer explained the importance of the scale and the procedure, read aloud each item, and performed a face-to-face interview without influencing the answers, in a quiet room and without interruptions. Apart from rating frequency, ratings of the disruptiveness of the observed behaviors were included, with questions examining the level of disruptiveness of each item to the staff according to the following grading: not at all; a little; moderately; very much; extremely. The corresponding numeric rating scale was as follows: 1 = never; 2 = less than once a week but still occurring; 3 = once or twice a week; 4 = several times a week; 5 = once or twice per d; 6 = several times per d; 7 = several times per h. The obtained scores reflected the average frequency of occurrence in the previous 2 weeks. NanoBEO was most effective at improving the frequency of occurrence of agitated behaviors at the first time point (Figure 4B), which corresponds to 2 weeks of treatment. Statistically significant differences were noted for all time points *versus* baseline for frequency (Figure 4C; time factor ****p < 0.0001; participant matching ****p < 0.0001; NanoBEO: 2, 4, and 6 weeks **p < 0.01; 8 weeks ***p < 0.001) and for disruptiveness (Figure 5C; time factor ****p < 0.0001; participant matching ****p < 0.0001; NanoBEO: 2 weeks **p < 0.01; 4, 6, and 8 weeks ***p < 0.001). A 28% improvement in the frequency of agitated behaviors was observed in the NanoBEO group in comparison with 6.93% in the placebo group. The observed rate was nearly as high as the threshold rate of 30% for improvement, which is generally regarded as significant in clinical trials that investigate the efficacy of interventions for the management of BPSD (Ballard et al., 2002); this is an important result, despite the underpowered study due to the pilot nature of the clinical trial. Individual data showed a homogeneous distribution, confirming the effectiveness of NanoBEO against agitation in most patients regarding the frequency and disruptiveness of behaviors. The effects were present in the entire 4-week treatment period for both factors. The effect on frequency gradually decreased during follow-up, while that on disruptiveness was maintained after the end of treatment and for the entire 4 weeks of follow-up.

### 3.3 Efficacy on pain

**FIGURE 6:**
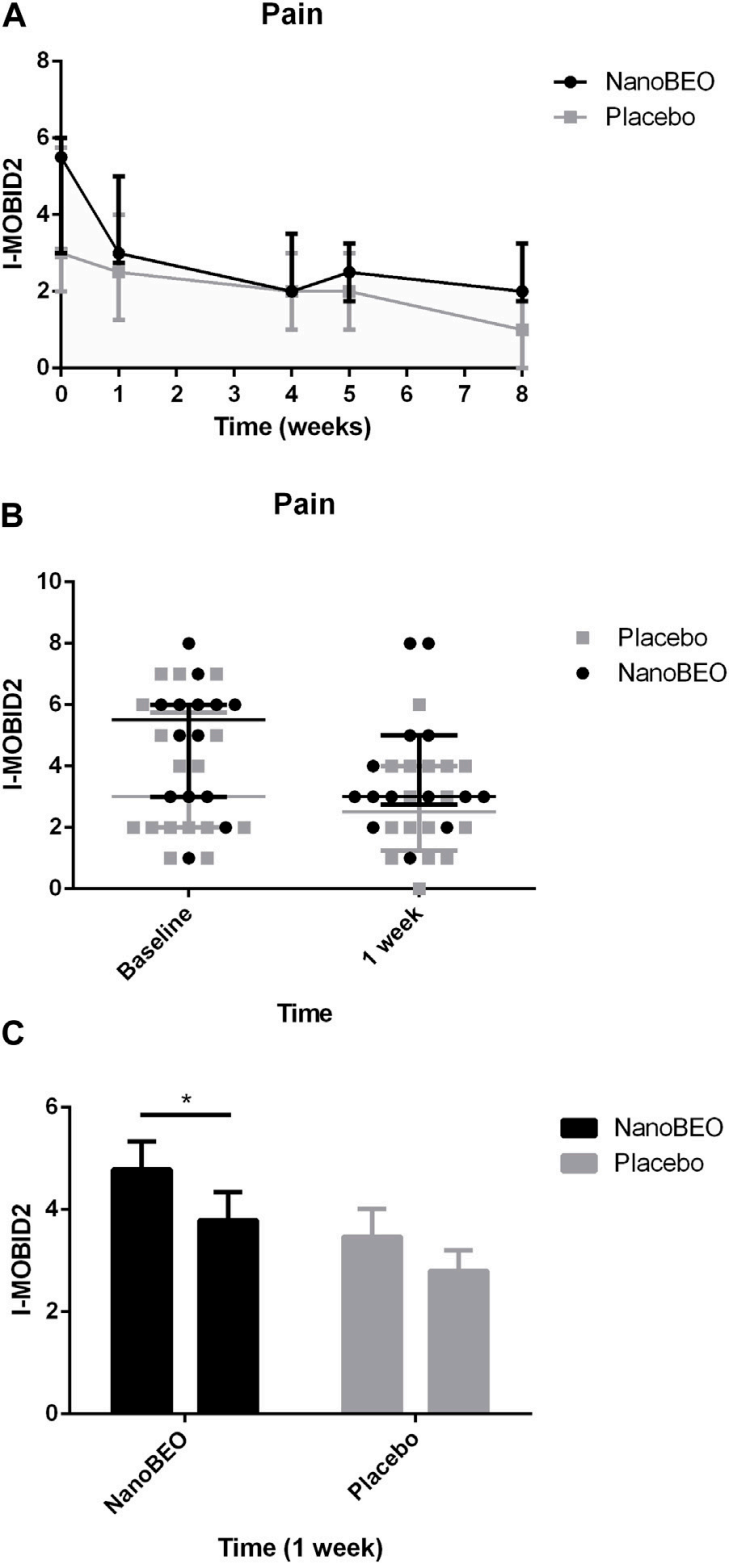
Efficacy of NanoBEO on pain based on the I-MOBID2 scores during treatment and follow-up **(A)** and as individual data at baseline and after 1 week of treatment **(B)**; data are expressed as the median + IQR. Patients allocated to the NanoBEO group exhibited decreased I-MOBID2 scores than did patients in the placebo group after 1 week; the scores decreased until the end of treatment and increased during the follow-up period but without reaching the baseline value. **(C)** Statistically significant differences in the effectiveness of NanoBEO (data are expressed as the mean ± SEM; time factor **p = 0.0031; participant matching ****p < 0.0001; NanoBEO baseline vs 1 week *p < 0.05). *p values <0.05 indicate statistical significance. n: NanoBEO = 14, placebo = 16.

The patients allocated to the NanoBEO group presented with higher pain intensity at baseline than the patients in the placebo group, as assessed by the I-MOBID2 scores, although this trend did not reach statistical significance (Figures 6A–C). These differences in the baseline values influenced the differences between the two arms throughout the study period. The I-MOBID2 pain scale was recently made available for the Italian nursing homes, after validation in a cohort of patients with AD with communication issues, aged 65 years and older and with an MMSE score ≤12 (Scuteri et al., 2022a). We selected I-MOBID2 because it is the only pain scale to consider co-occurrence of musculoskeletal and visceral pain (Hadjistavropoulos et al., 2007) and to allow examination of even hidden pain conditions through active, guided movements (Husebo et al., 2010). The I-MOBID2 has demonstrated good face and content validity (0.89), high construct validity (Spearman rank-order correlation Rho = 0.748), reliable internal consistency (Cronbach’s α coefficient = 0.751), good-to-excellent inter-rater (intraclass correlation coefficient [ICC] = 0.778) and test–retest (ICC = 0.902) reliability, and good inter-rater and test–retest agreement (Cohen’s K = 0.744) with short training and average execution time of 5.8 min (Scuteri et al., 2022a). Hence, we obtained all relevant data using a tool that allowed assessment in the absence of confounding bias attributed to the influence of stressors on the patients. For pain as secondary endpoint, the results after 1 week of treatment demonstrated that NanoBEO had a statistically significant analgesic efficacy in comparison with the placebo (Figure 6C), even in our heterogeneous sample of patients regarding pain intensity. The data after the entire 4-week treatment with NanoBEO demonstrated increased effectiveness, with the reported intensity reduced up to half of that at baseline (Figures 6A–C). In particular, the improvement in pain intensity after the first week of treatment reached 45.46% compared with 16.67% of the placebo. The observed rate exceeded the threshold rate of 30% for improvement; this value generally reflects clinically important changes in clinical trials that investigate the efficacy of interventions for pain management, according to the recommendations of the Initiative on Methods, Measurement, and Pain Assessment in Clinical Trials (IMMPACT) (Dworkin et al., 2008). After 1 week of follow-up (week 5) and at the end of the entire observational period (week 8), pain intensity in the NanoBEO group progressively returned to the level after 1 week of treatment, although differences were not statistically significant (Figure 6A).

### 3.4 Safety

Treatment with NanoBEO was well tolerated and no patient discontinued the trial because of adverse reactions related to the application. No side effects were reported at any of the 4-week assessments and during the follow-up period. The biochemical analyses were not affected by the NanoBEO treatment, as presented in Tables 3A–E for the main parameters.

**TABLE 3:** Assessment of biochemical parameters—azotemia (a), and levels of serum creatinine (b), creatine phosphokinase (CPK; c), and transaminases (d, e)—at baseline, after 4-week treatment with NanoBEO and at the end of the follow-up period. The reference values, depending on the laboratory, are reported in brackets.

Azotemia (mg/dL)			
Patient ID	Baseline	Treatment (week 4)	Follow-up (week 8)
D02	42.00 (17.00–43.00)	53.00 (17.00–43.00)	46.7 (17.00–43.00)
D04	74.00 (17.00–43.00)	111.00 (17.00–43.00)	82.9 (17.00–43.00)
D06	77.00 (17.00–43.00)	54.00 (17.00–43.00)	74.00 (17.00–43.00)
D08	39.00 (17.00–43.00)	46.00 (17.00–43.00)	32.00 (17.00–43.00)
D13	____	95.00 (17.00–43.00)	79.00 (17.00–43.00)
D14	29.00 (17.00–43.00)	41.00 (17.00–43.00)	66.00 (17.00–43.00)
D16	48.00 (10.00–55.00)	41.00 (10.00–55.00)	50.00 (10.00–55.00)
D19	61.00 (10.00–55.00)	45.00 (10.00–55.00)	46.00 (10.00–55.00)
D20	78.00 (10.00–55.00)	95.00 (10.00–55.00)	110.00 (10.00–55.00)
D22	35.00 (17.00–43,00)	33.00 (17.00–43.00)	34,3 (17.00–43.00)
D23	55.00 (17.00–43.00)	31.00 (17.00–43.00)	49.1 (17.00–43.00)
D27	41.00 (20.00–50.00)	29.00 (10.00–50.00)	35.00 (10.00–50.00)
D28	49.00 (10.00–50.00)	53.00 (10.00–50.00)	58.00 (10.00–50.00)
D31	37.00 (10.00–50.00)	44.00 (10.00–50.00)	37.00 (10.00–50.00)

Serum creatinine (mg/dL)			
Patient ID	Baseline	Treatment (week 4)	Follow-up (week 8)
D02	0.83 (0.66–1.09)	0.92 (0.66–1.09)	0.87 (0.66–1.09)
D04	1.4 (0.66–1.09)	1.62 (0.66–1.09)	1.48 (0.66–1.09)
D06	1.23 (0.66–1.09)	1.15 (0.66–1.09)	1.14 (0.66–1.09)
D08	0.93 (0.66–1.09)	1.09 (0.66–1.09)	0.81 (0.66–1.09)
D13	____	1.7 (0.66–1.09)	1.77 (0.66–1.09)
D14	0.78 (0.66–1.09)	0.9 (0.66–1.09)	0.9 (0.66–1.09)
D16	0.88 (0.60–1.30)	0.84 (0.50–0.90)	0.84 (0.50–0.90)
D19	0.95 (0.60–1.30)	0.82 (0.50–0.90)	0.89 (0.50–0.90)
D20	1.42 (0.60–1.30)	1.40 (0.50–0.90)	1.63 (0.50–0.90)
D22	0.64 (0.66–1.09)	0.68 (0.66–1.09)	0.77 (0.66–1.09)
D23	0.84 (0.66–1.09)	0.74 (0.66–1.09)	0.95 (0.66–1.09)
D27	1.19 (0.50–0.90)	0.99 (0.60–1.30)	1.14 (0.60–1.30)
D28	0.59 (0.70–1.40)	0.68 (0.70–1.40)	0.57 (0.70–1.40)
D31	0.73 (0.60–1.30)	0.77 (0.60–1.30)	0.67 (0.60–1.30)

Creatine phosphokinase (CPK; IU)			
Patient ID	Baseline	Treatment (week 4)	Follow-up (week 8)
D02	42 (0–145)	59 (0–145)	66 (0–145)
D04	28 (0–145)	20 (0–145)	19 (0–145)
D06	58 (0–145)	27 (0–145)	31 (0–145)
D08	19 (0–145)	25 (0–145)	20 (0–145)
D13	93 (0–145)	88 (0–145)	100 (0–145)
D14	30 (0–145)	35 (0–145)	73 (0–145)
D16	____	____	____
D19	77 (26–192)	57 (26–192)	49 (26–192)
D20	42 (26–192)	34 (26–192)	44 (26–192)
D22	30 (0–145)	40 (0–145)	25 (0–145)
D23	46 (0–145)	50 (0–145)	53 (0–145)
D27	____	16 (34–145)	15 (34–145)
D28	47 (46–171)	46 (46–171)	39 (46–171)
D31	36 (34–145)	15 (34–145)	15 (34–145)

Serum GOT transaminase (IU)			
Patient ID	Baseline	Treatment (week 4)	Follow-up (week 8)
D02	20 (11–34)	23 (11–34)	17 (11–34)
D04	15 (11–34)	16 (11–34)	11 (11–34)
D06	16 (11–34)	17 (11–34)	18 (11–34)
D08	16 (11–34)	17 (11–34)	15 (11–34)
D13	____	39 (11–34)	40 (11–34)
D14	10 (11–34)	16 (11–34)	65 (11–34)
D16	21 (0–33)	14 (0–33)	15 (0–33)
D19	16 (0–33)	12 (0–33)	11 (0–33)
D20	41 (0–33)	31 (0–33)	28 (0–33)
D22	12 (11–34)	13 (11–34)	11 (11–34)
D23	19 (11–34)	30 (11–34)	20 (11–34)
D27	16 (0–31)	22 (5–38)	12 (5–38)
D28	13 (5–38)	21 (5–38)	12 (5–38)
D31	28 (5–38)	40 (5–38)	18 (5–38)

Serum GPT transaminase (IU)			
Patient ID	Baseline	Treatment (week 4)	Follow-up (week 8)
D02	11 (7–41)	14 (7–41)	9 (7–41)
D04	4 (7–41)	4 (7–41)	3 (7–41)
D06	9 (7–41)	12 (7–41)	10 (7–41)
D08	13 (7–41)	9 (7–41)	7 (7–41)
D13	____	21 (7–41)	24 (7–41)
D14	9 (7–41)	15 (7–41)	14 (7–41)
D16	7 (0–32)	8 (0–32)	16 (0–32)
D19	9 (0–32)	11 (0–32)	10 (0–32)
D20	19 (0–32)	14 (0–32)	10 (0–32)
D22	7 (7–41)	10 (7–41)	7 (7–41)
D23	7 (7–41)	23 (7–41)	8 (7–41)
D27	10 (0–31)	____	15 (0–49)
D28	12 (0–49)	23 (0–49)	14 (0–49)
D31	34 (0–49)	45 (0–49)	19 (0–49)

GPT: glutamic-pyruvic transaminase (also known as alanine aminotransferase [ALT]).

GOT: glutamic-oxaloacetic transaminase (also known as aspartate aminotransferase [AST]).

## 4 Discussion

Although BPSD were initially considered in relation to cognitive decline, their high prevalence suggests that they are important markers for the prognosis of dementia and determinants of quality of life (Cerejeira et al., 2012). In the complex framework of lack of disease-modifying therapies for all disease stages and universally efficacious, recent pharmacological approaches have focused on cannabinoids. However, these should be reserved for PwD who do not present with significant cardiovascular disease, which is common in this population because of age-related comorbidities (Scuteri et al., 2022b). The lack of effective and safe treatments for the management of agitation, one of the most challenging BPSD for clinicians and caregivers, prompted the identification of symptom-specific, patient-centered, non-pharmacological interventions, which target the needs that may trigger the behavioral disorder (Eunhee et al., 2023). This aspect is of utmost importance, particularly for the steadily growing population of older PwD who are subjected to polydrug therapies for chronic medical conditions other than dementia (at rates higher than those for cognitively intact counterparts) (Nørgaard et al., 2017; Growdon et al., 2021); a non-pharmacological approach may be a safe option to avoid drug interactions (Letinier et al., 2022) and increased adverse effects due to inappropriate prescriptions (Rodrigues and Oliveira, 2016) or disrupted metabolism and elimination processes (Mangoni and Jackson, 2004; McLachlan et al., 2009). Furthermore, deprescribing is fundamental, considering that these patients are excluded from clinical trials for pain conditions unrelated to dementia, thus leading to unpredictable adverse reactions (Scuteri et al., 2022e; Scuteri et al., 2022f). PwD in advanced stages and older than 65 years are reported to receive five or more medications of which, in 39% of cases, at least one is potentially inappropriate according to the Beers Criteria (Riedl et al., 2022).

As the available pharmacological treatments have limited efficacy and considerable side effects, the present pilot clinical trial investigated the efficacy and safety of a non-pharmacological device based on an essential oil with anxiolytic-like activity and, different from benzodiazepines, devoid of sedative properties. Furthermore, according to the preclinical evidence generated on BEO, its combination with morphine enhanced the anti-allodynic effect (Kuwahata et al., 2013) and, in the formalin test, pretreatment with the opioid receptor antagonist naloxone methiodide, not able to cross blood brain barrier, attenuated the effect of BEO, thus suggesting potential involvement of peripheral opioid mechanisms (Katsuyama et al., 2015). Low concentrations of BEO can induce the exocytosis of glutamate that modulates pain through mGluRs, involved in the release of endogenous opioid peptides and endocannabinoids with analgesic activity (Scuteri et al., 2019). Twenty-nine patients (96.67%) completed the trial (treatment period and follow-up). A decrease in the frequency and disruptiveness of agitated behaviors was demonstrated in the NanoBEO group compared with that of the placebo group. NanoBEO was most effective after 2 weeks of treatment, and improvement in the frequency of agitated behaviors reached 28% compared with 6.93% in the placebo group; the rate is nearly up to the threshold rate of 30% improvement that is generally regarded as clinically significant in trials on the efficacy of interventions for the management of BPSD (Ballard et al., 2002). This result is noteworthy, even more so when considering that this was an underpowered pilot clinical trial. Additionally, our finding is important in view of the high placebo response rates registered in this context (Ballard and O'Brien, 1999; Ballard et al., 2002) and of the modest effectiveness of neuroleptics in reducing symptoms (Schneider et al., 1990). The individual data displayed a homogeneous distribution that supports the effectiveness of the treatment on agitation in most patients regarding the frequency and disruptiveness of agitated behavior, as measured using CMAI. The efficacy of NanoBEO on agitated behaviors was observed for the entire 4-week treatment period for both variables. The effect on frequency gradually decreased during the follow-up period, while that on disruptiveness was retained after the end of the treatment and for the entire 4-week follow-up period. Interestingly, no additional psychotropic drugs were prescribed as rescue medication for agitation during the study, strengthening the efficacy of NanoBEO in prolonged therapy of advanced dementia.

The Describe–Investigate–Create–Evaluate (DICE) model suggests that BPSD is caused by disruptions in brain circuitries that predispose to enhanced vulnerability to triggers such as pain; accordingly, assessment and treatment are fundamental to handling symptoms such as agitation (Kales et al., 2014; 2015; 2019a; Kales et al., 2019b). Supporting evidence demonstrates that older adults require treatment for at least 6 months to alleviate chronic pain (Blyth et al., 2001), with unsuccessful outcomes in approximately 80% of the cases (American Geriatrics Society Panel on the Pharmacological Management of Persistent Pain in Older, 2009). Undertreated pain remarkably affects the Italian population. This was demonstrated by the Italian Silver Network Home Care project, according to which, of the approximately 49% of the patients who experience daily pain, only 25% receive a World Health Organization (WHO) I level analgesic (Landi et al., 2001). In this context, the purpose of the present clinical trial was to shed light on the possible increased efficacy of NanoBEO in PwD attributed to its double effect on both the primary endpoint of agitation and the secondary endpoint of pain. The results after 1 week of treatment confirmed the significant analgesic efficacy of NanoBEO compared with that of the placebo. The data after completion of the entire intervention with NanoBEO demonstrated increased effectiveness, with decreased levels of pain intensity up to approximately half of that at baseline. After 1 week of follow-up (week 5) and at the end of the observation period (week 8), the level of pain intensity in the NanoBEO group progressively returned to the level recorded after 1 week of treatment. The improvement in pain intensity, as measured by I-MOBID2, after the first week of treatment was 45.46% compared to 16.67% of the placebo. The recorded rate exceeded the threshold of 30% for clinically important rates in clinical trials on the efficacy of interventions for pain management according to IMMPACT recommendations (Dworkin et al., 2008).

The limitations of this study include the small sample size because of the pilot nature of the trial, the difference in pain intensity at baseline in the two groups, and flaws in two dispensers (one each in the NanoBEO and placebo groups), which were replaced. The treatment with NanoBEO was well tolerated and no patient discontinued the trial because of adverse reactions related to the treatment. No side effects were reported at any of the 4-week assessments and during follow-up. Additionally, the biochemical analyses (azotemia, and serum creatinine, CPK, and transaminase levels) were not influenced by the treatment with NanoBEO, demonstrating the safety of the product.

This pilot study supplied substantial evidence for the subsequent large-scale pivotal trial that will address all present limitations; we expect to confirm these promising results and will investigate the efficacy and safety of aromatherapy using a rigorous blinded design adequately powered to allow the clinical translation of NanoBEO.

## Figures and Tables

**Table:** 

**AD**	Alzheimer’s disease
**ALT**	alanine aminotransferase
**aMCI**	amnestic mild cognitive impairment
**AST**	aspartate aminotransferase
**Aβ**	β-amyloid
**BEO**	bergamot essential oil
**BPSD**	behavioral and psychological symptoms of dementia
**CI**	confidence intervals
**CMAI**	Cohen-Mansfield Agitation Inventory
**CONSORT**	Consolidated Standards of Reporting Trials
**CPK**	creatine phosphokinase
**DICE**	Describe–Investigate–Create–Evaluate
**EMA**	European Medicine Agency
**FDA**	Food and Drug Administration
**GOT**	glutamic-oxaloacetic transaminase
**GPT**	glutamic-pyruvic transaminase
**HMPC**	Committee on Herbal Medicinal Products
**ICC**	intraclass correlation coefficient
**IMMPACT**	Initiative on Methods, Measurement, and Pain Assessment in Clinical Trials
**I-MOBID2**	Italian Mobilization–Observation–Behavior–Intensity–Dementia
**IQR**	interquartile range
**MBI**	mild behavioral impairment
**MMSE**	mini‐mental state examination
**MRI**	magnetic resonance imaging
**MWU**	Mann–Whitney *U* test
**PwD**	people with dementia
**SAP**	statistical analysis plan
**SD**	standard deviation
**SEM**	standard error of the mean
**SPECT**	single-photon emission computed tomography
**SPIRIT**	Standard Protocol Items: Recommendations for Interventional Trials
**WHO**	World Health Organization

## Data Availability

The original contributions presented in the study are included in the article/Supplementary Material, further inquiries can be directed to the corresponding author.
