# Supplementary material for: Efficacy of therapeutic intervention with NanoBEO to manage agitation and pain in patients suffering from severe dementia: a pilot clinical trial
Source: Front Pharmacol. 2024 Aug 1;15:1417851. doi: 10.3389/fphar.2024.1417851 (PMC11325727; doi:10.3389/fphar.2024.1417851)
Supplement: Supplementary file 1 [file DataSheet1.docx]

**List of the Pilot BRAINAID trial investigators and sites**

1. CASA DI RIPOSO S. MARIA, via Salvatore Andreoli 88835 Roccabernarda (KR),

Dr Teresa Stumpo

2. VILLA SAN FRANCESCO, via Catanzaro 88835 Roccabernarda (KR),

Dr Giuseppina Schipani

3. RSA PADRE GIUSEPPE MOSCATI, via III Fontane 31, 88054 Sersale (CZ),

Dr Sabrina Tomaselli

4. RSA S. ANNA, via Enrico Berlinguer 52, 88070 Botricello (CZ),

Dr Domenico D’Elia

5. RSA SANTA RITA, via San Paolo 22, 88838 Mesoraca (KR),

Dr Tommasina Lavigna

6. CASA GIARDINO OLIVETI, via Laghi Silani 339, 88836 Cotronei (KR),

Dr Teresa Loria
